# Post-radiation sarcoma of the neck treated with re-irradiation followed by wide excision

**DOI:** 10.1186/1477-7819-4-69

**Published:** 2006-10-04

**Authors:** Alex Tan, Samuel YK Ngan, Peter FM Choong

**Affiliations:** 1 Sarcoma Unit, Peter MacCallum Cancer Centre, Melbourne, Australia; 2 The University of Melbourne, Parkville, Australia; 3 Department of Orthopedic Surgery, St Vincent's Hospital, Melbourne, Australia

## Abstract

**Background:**

Post-radiation sarcoma (PRS) is an uncommon disease manifesting as sarcoma in a previously irradiated field, usually with a latent period of 5 years or more. Literature is limited to small series. Optimal management of this disease is unclear. Positive margins are common following attempted curative surgery and outcomes are poor. Radiotherapy is hardly used and its effect on PRS is not known. We described a case of PRS treated with preoperative radiotherapy followed by margin-negative wide excision.

**Case presentation:**

The 59-year-old patient presented with a mass in the left supraclavicular fossa and numbness in the arm, six years following radical irradiation of the head and neck for nasopharyngeal carcinoma. Open biopsy showed pleomorphic spindle cell sarcoma. She was treated with pre-operative hyperfractionated radiotherapy followed by margin-negative wide excision and nerve grafting. Cumulative radiation dose to the supraclavicular fossa was 98 Gy. Histological examination of the post-irradiation tumor specimens showed evidence of significant tumor response to re-irradiation. The patient remained free of disease five years after surgery with excellent functional outcome.

**Conclusion:**

Role of radiotherapy in PRS is uncertain. We described a case that was successfully managed with preoperative radiotherapy and margin-negative wide excision in terms of tumor control and functional outcomes. The impact of radiotherapy was demonstrated in the post-irradiation resected specimen. Further investigation using re-irradiation and surgery in PRS is warranted.

## Background

Post-radiation sarcoma (PRS) is an uncommon disease. It occurs in approximately 0.2% of patients treated with radiotherapy that survive at least five years [1], with a wide latent period between initial irradiation and development of sarcoma ranging from 4–55 years [2]. Outcomes are generally poor: 5-year overall survival figures of 11 – 29% have been quoted [3,4] for all groups and for those who have a curative resection the 5 year survival is approximately 40% [5]. Survival figures are worse for proximally situated tumors [6]. Poor survival is multifactorial: margin-negative surgery is often difficult as tumors are more likely to be large [7], and there is a high-risk of distant metastasis.

Standard treatment for PRS is radical resection. There is a paucity of chemotherapeutic agents that have shown efficacy in the management of PRS [6]. The role of adjuvant therapy has not been defined. Although radiation therapy has an established role in the management of soft tissue sarcoma [8,9], it is seldom used in PRS. The reason for this is usually multifactorial. The normal tissues surrounding the PRS have usually been irradiated to near-tolerance radiation doses during the initial radiation therapy. The efficacy of radiation in PRS is uncertain. There is also the potential risk of inducing another malignancy.

In this report, we described the case of a patient with PRS involving the supraclavicular fossa and brachial plexus following successful treatment of nasopharyngeal carcinoma with radiotherapy five years earlier. Preoperative accelerated hyperfractionated radiotherapy was used followed by *en bloc *resection and nerve grafting, leaving the patient with an excellent functional outcome and free of disease five years following surgery.

## Case presentation

The patient, a 59-year-old Chinese woman, was treated at our centre in 1994 for a T3N0M0 nasopharyngeal carcinoma. She was treated with external beam radiotherapy to 60 Gy in 30 fractions over 6 weeks in accordance to ICRU-50. Two lateral parallel opposed fields were used to treat the nasopharynx and upper neck. The lower neck was irradiated with a direct anterior field with a central cord shield and a lower border just below clavicular heads. The junction of the upper and lower field was at the level of hyoid bone. It was followed by a 10 Gy Iridium-192 brachytherapy boost to the nasopharynx. Treatment was completed in May 1994. She remained well with no further recurrence of the nasopharyngeal carcinoma but developed xerostomia and mild fibrosis of the lower neck.

**Figure 1:**
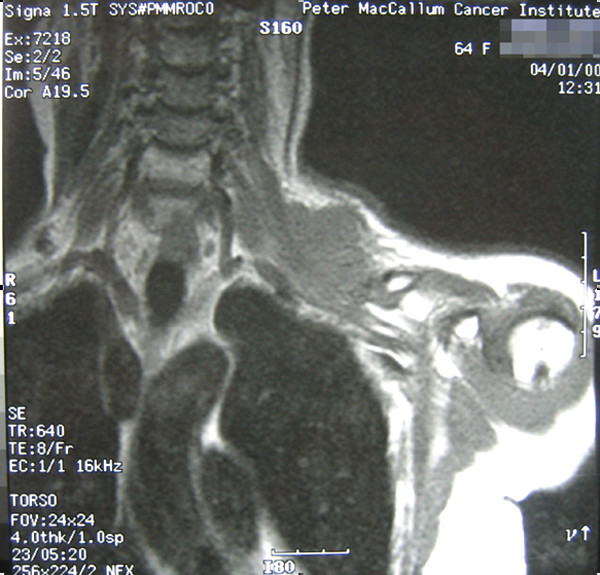
MRI showing soft tissue mass involving the upper branches of the brachial plexus.

She re-presented in December 1999 with a mass in the left supraclavicular fossa and numbness in the arm, but no motor weakness. The mass was located within the area previously irradiated to 60 Gy. Her disease was staged with our standard protocol at the time with MRI of the neck and shoulder, CT of the chest and functional imaging using thallium-201 scintigraphy [10]. MRI showed a 3 cm × 5 cm mass involving the brachial plexus (Figure 1). Open biopsy confirmed pleomorphic spindle cell sarcoma.

The management of her case was discussed in our multi-disciplinary meeting. The agreed treatment plan was preoperative radiotherapy followed by *en bloc *resection. She received an accelerated hyperfractionated course of radiotherapy 48 Gy in 40 fractions (ICRU-50), treating twice a day over four weeks. The radiation field incorporated the brachial plexus and used 6 MV photons in an anterior-weighted, opposed oblique arrangement avoiding the spinal cord. Radiotherapy was completed in February 2000. Restaging investigations showed that the lesion had become more cystic on MRI, but its size had not changed. The degree of thallium-201 scintigraphy uptake had not changed significantly either. The patient proceeded on to surgery four weeks later.

Surgery in March 2000 consisted of *en bloc *resection of the tumor along with the left sternomastoid and superior and middle trunks of the brachial plexus. Clavicular osteotomy was necessary to allow dissection of the tumor from the subclavian vessels. Sural nerve grafts were used to restore continuity of nerve supply from C5 and C6 to the suprascapular, musculocutaneous and axillary nerves. A latissimus dorsi myocutaneous flap was raised to close the defect in the neck. The middle third of the clavicle that had been reflected downwards was replaced and secured with a plate and screws.

**Figure 2:**
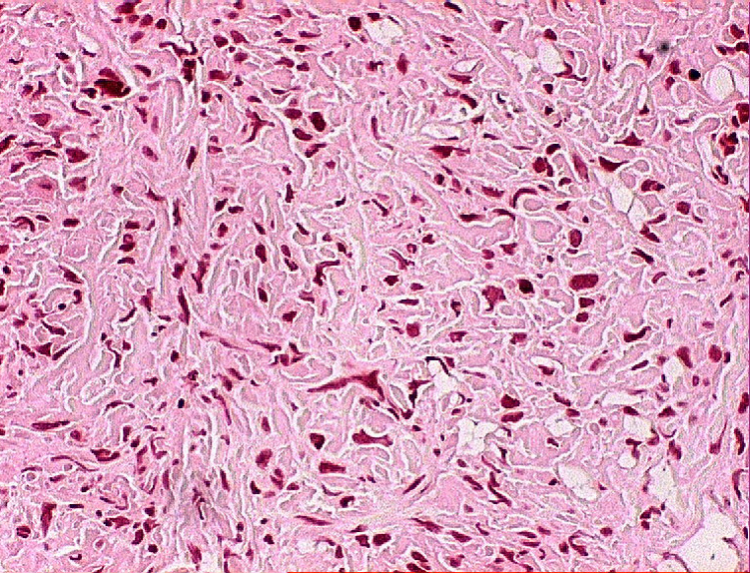
The open biopsy showing pleomorphic spindle cell sarcoma.

**Figure 3:**
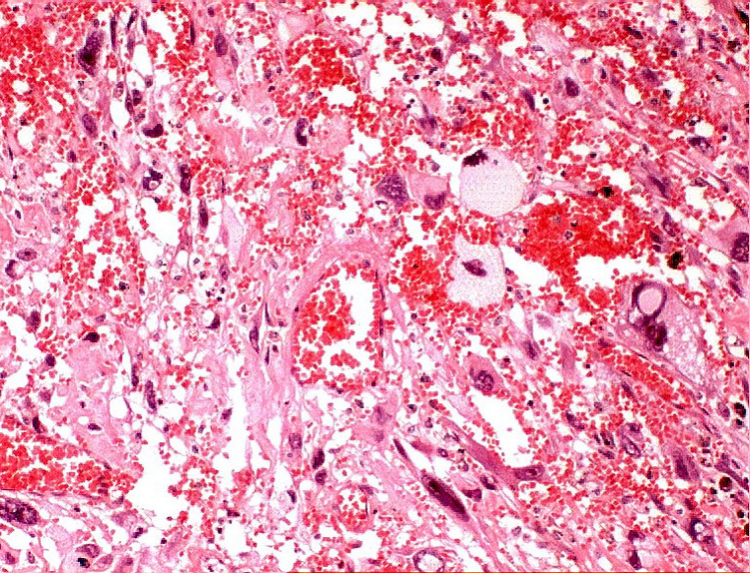
The surgical specimen showing areas of necrosis and hemorrhage following radiotherapy.

Histological examination of the surgical specimen showed evidence of radiotherapy, with large areas of necrosis and hemorrhage seen both macroscopically and microscopically. Comparison was made between the operative specimen and the pre-irradiation biopsy; necrosis was not seen in the pre-irradiation specimen (Figures 2 and 3). Surgical excision appeared complete.

Immediate postoperative recovery was uncomplicated. The patient retained good function in her hand but was left with a flail shoulder and no power in elbow flexion. She thus underwent cable grafting of the musculocutaneous nerve in October 2000, with gradual return of elbow function following this procedure.

**Figure 4:**
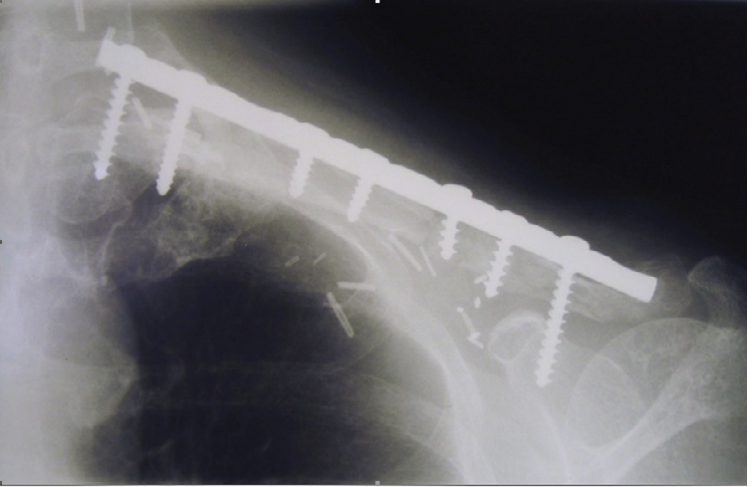
Alignment of the clavicle following bone grafting and internal fixation.

The patient presented again in December 2001 with pain in the shoulder. X-ray confirmed that there had been non-union of the osteopaenic clavicle, the plate having detached from the lateral end of the clavicle. She returned to theatre and had a bone graft and internal fixation (Figure 4). Following this procedure, pain settled and she continues to have good use of her arm, although shoulder movement remains minimal.

The patient remains well five years after combined therapy for the PRS, with no evidence of recurrence of either her nasopharyngeal carcinoma or the PRS. The patient had no lymphoedema, neurovascular, severe fibrosis or other significant late effect as a result of re-irradiation.

## Discussion

This case demonstrates that PRS can be effectively managed with preoperative radiotherapy and surgery, providing excellent long-term control without necessarily sacrificing function. The intent of radiotherapy was to sterilize the microscopic disease at the periphery of the PRS in order to maximize the chance of local control, as the extent of the surgical resection was limited by the anatomy of the supraclavicular fossa.

Complete resection rates in PRS are relatively low; approximately 50% of patients undergoing attempted curative resection are left with either macroscopic or microscopic residual disease [5]. Even with clear margins, local recurrence rates are approximately 50% in small series [6]. In this case, preoperative radiotherapy was given to try to increase the likelihood of margin-negative surgery, especially given the difficulty in achieving wide surgical margins in a region such as the supraclavicular fossa, which is densely packed with major vessels and nerves. If preoperative radiotherapy had been omitted and margins at surgery are close or positive, postoperative radiotherapy would have been necessary to achieve long-term control. This would have necessitated a higher total dose and larger volume than in the preoperative setting for the same outcome [8], and would result in greater incidence of long-term toxicity such as fibrosis, oedema and joint stiffness than pre-operative radiation [11].

The brachial plexus was one of the dose-limiting structures at risk of late effects following re-irradiation. The dose of 48 Gy was delivered in fraction sizes of 1.2 Gy: biologically equivalent to 38 Gy in 2 Gy fractions (using an α:β ratio of 2) and thus a total dose to the brachial plexus of 98 Gy – considerably higher than the TD_5/5 _tolerance dose of the brachial plexus, which is approximately 62 Gy [12]. However the planned and necessary curative surgery involved sacrifice of two of the three trunks of the brachial plexus and immediate sural nerve grafting. The development of radiation neuropathy was therefore considered an acceptable risk as many of the structures at risk were to be resected along with the tumor.

Histological examination of the surgical specimen showed that there were large areas of necrosis and hemorrhage in the specimen, which were not evident on review of the pre-irradiation open biopsy. Although the response of PRS to radiation has not been widely studied, it is traditionally thought to be relatively insensitive to radiation. This histological finding suggests that the radiotherapy had a significant tumoricidal effect and may have enhanced the surgeon's ability to achieve clear surgical margins despite the difficult location of the tumor. It may also have sterilized subclinical areas of disease adjacent to the tumor, contributing to her long-term disease control.

There was non-union of the osteopaenic clavicle following her initial surgery, which culminated in separation of the plate from the lateral end of the clavicle and bearing of the arm's weight by the rotator cuff muscles, which then resulted in pain and spasm. Pathologic fracture and fracture non-union is well described in the post-radiotherapy setting [13] and is thought to be related to injury to osteoprogenitor cells, damage to Haversian canals and reduction in new vessel formation at the fracture site [14]. Successful repair of the fracture was achieved by bone grafting and internal fixation.

## Conclusion

Role of radiotherapy in PRS is uncertain. We described a case that was successfully managed with preoperative radiotherapy and margin-negative wide excision in terms of tumor control and functional outcomes. The impact of radiotherapy was demonstrated in the post-irradiation resected specimen. Further investigation using re-irradiation and surgery in PRS is warranted.

## Competing interests

The author(s) declare that they have no competing interests.

## Authors' contributions

**AT **reviewed the literature and prepared the manuscript. **SN **and **PC **assisted in manuscript revision and review. All authors read and approved the final manuscript.
